# The effects of *Ascophyllum nodosum, Camellia sinensis-leaf* extract, and their joint interventions on glycolipid and energy metabolism in obese mice

**DOI:** 10.3389/fnut.2023.1242157

**Published:** 2023-08-24

**Authors:** Yuhan Xu, Xiuzhen Jia, Wei Zhang, Qiaoling Xie, Meizhen Zhu, Zifu Zhao, Jingyu Hao, Haoqiu Li, Jinrui Du, Yan Liu, Haotian Feng, Jian He, Hongwei Li

**Affiliations:** ^1^State Key Laboratory of Vaccines for Infectious Diseases, Xiang An Biomedicine Laboratory, State Key Laboratory of Molecular Vaccinology and Molecular Diagnostics, National Innovation Platform for Industry-Education Integration in Vaccine Research, School of Public Health, Xiamen University, Xiamen, China; ^2^School of Public Health, Xiamen University, Xiamen, China; ^3^Inner Mongolia Dairy Technology Research Institute Co., Ltd., Hohhot, China; ^4^Yili Innovation Center, Inner Mongolia Yili Industrial Group Co., Ltd., Hohhot, China

**Keywords:** obesity, seaweed extract, green tea extract, respiratory rhythm, intestinal flora, glycolipid metabolism

## Abstract

**Objectives:**

Obesity is often associated with glucolipid and/or energy metabolism disorders. *Ascophyllum nodosum* extract (seaweed extract, SE) and *Camellia sinensis-leaf* extract (tea extract, TE) have been reported to promote positive metabolic effects through different mechanisms. We investigated the effects of SE and TE on metabolic homeostasis in diet-induced obese mice and discussed their functional characteristics.

**Methods:**

Male C57BL/6J mice fed with high-fat diets for 8 weeks were established as obese models and subsequently divided into different intervention groups, followed by SE, TE, and their joint interventions for 10 weeks. Body weight and food intake were monitored. Fasting glucose and oral glucose tolerance tests were interspersed during the experiment. After the intervention, the effects on obesity control were assessed based on body composition, liver pathology section, blood lipids and glucose, respiratory exchange ratio (RER), energy expenditure (EE_1_, EE_2_, and EE_3_), inflammatory factors, lipid anabolism enzymes, and gut flora of the obese mice.

**Results:**

After continuous gavage intervention, the mice in the intervention groups exhibited lower body weight (lower ~4.93 g, vs. HFD 38.02 g), peri-testicular fat masses (lower ~0.61 g, vs. HFD 1.92 g), and perirenal fat masses (lower ~0.21 g, vs. HFD mice 0.70 g). All interventions prevented diet-induced increases in plasma levels of glucose, adiponectin, leptin, and the inflammatory factors IL-1β and TNF-α. The RER was modified by the interventions, while the rhythm of the RER was not. Blood lipids (total cholesterol, triglycerides, and LDL) decreased and were associated with lower lipid anabolism enzymes. In addition, the SE and TE interventions altered the structure and abundance of specific flora. Different interventions inhibited the growth of different genera positively associated with obesity (*Escherichia–Shigella, Helicobacter*, etc.) and promoted the growth of *Akkermansia* and *Bacteroides*, thus affecting the chronic inflammatory state.

**Conclusion:**

SE and TE both have synergistic effects on weight control and glucolipid metabolism regulation by improving insulin sensitivity and reducing lipid synthesis-related enzyme expression, whereas the combination of SE and TE (3:1) has a better effect on regulating energy metabolism and inhibiting chronic inflammation.

## 1. Introduction

Economic and social development has facilitated people's lives. However, uplifting their lifestyles has also exposed them to obesity. Obesity is a chronic metabolic disease wherein the body deviates from orderly energy homeostasis due to impaired fat accumulation (1). The World Health Organization (WHO) considers obesity a risk factor for various types of non-communicable diseases (NCDs) (2), accounting for ~2.8 million deaths from NCDs in 2021, including cardiovascular disease, diabetes, cancer, neurological disorders, chronic respiratory diseases, and digestive disorders. Aside from the health implications, obesity also has a serious economic impact, accounting for over 13% of all healthcare expenditures globally to the tune of 990 billion USD per year (3). The latest 2022 report showed that overweight and obesity prevalence has increased across all age groups and will continue to increase over the next decade (4).

A range of different pharmacotherapies have been developed for obesity in clinical practice; however, obesity's complex and diverse pathophysiological mechanisms render single-route pharmacological interventions often incomprehensible. Thus, an appropriate option to tackle multifactorial fat accumulation and obesity is to enable the simultaneous regulation of impaired metabolic pathways in multiple organs.

Marine organisms are rich sources of various bioactive substances (5). Seaweed extracts have a unique biochemical composition rich in various nutrients and bioactive substances. Shin et al. (6) observed a reduction in both total and low-density lipoprotein (LDL) cholesterol levels in patients with hypercholesterolemia after daily brown seaweed consumption. The correlation between several inflammatory biomarkers and insulin resistance suggested that an increase in pro-inflammatory factor inhibition could be a mechanism responsible for the effects of seaweed extracts on glucose homeostasis (7). *Ascophyllum nodosum* is a species of brown seaweed in the family *Fucaceae* rich in unique natural molecules. *Ascophyllum nodosum* extract (a seaweed extract, SE) processed through water extraction, ultrafiltration, spray drying, etc. has at least 35% phlorotannin content. Phlorotannin inhibits pro-inflammatory cytokine expression and interferes with transcriptional regulation (8). The study also provides evidence that phlorotannin-rich extracts hold potential for the management of the activity of α-glucosidase, α-amylase, and pancreatic lipase linked to metabolic disorders (9).

Epidemiologically, tea consumption might contribute to weight reduction and metabolic homeostasis improvement (10–12). Various flavonoids in tea, such as quercetin, kaempferol, myricetin, flavan-3-ols, and theaflavins, have been shown to have anti-obesity effects (11). The two main flavan-3-ols, namely epigallocatechin gallate (EGCG) and catechins, have been shown to reduce diet-induced obesity by inhibiting adipogenesis and reducing leptin levels and energy absorption (13). Furthermore, it was suggested that *Camellia sinensis-leaf* extract (a tea extract, TE) could promote hepatic fatty acid oxidation and reduce lipogenic enzyme expression (14).

Both *Ascophyllum nodosum* and *Camellia sinensis-leaf* extracts can regulate glycolipid metabolism, yet their modes of action differ. In terms of achieving energy homeostasis, we concluded that SE focuses on inhibiting chronic inflammation and/or regulating blood glucose and blood lipids, while TE focuses on energy regulation by adjusting the expression of cytokines and/or hormones. Obesity is originally a complex etiological process. Multi-pathway regulation is more promising for achieving a full range of obesity control. We not only wondered about the specific characteristics of the effects of each of SE and TE but also wished to explore the synergistic weight loss effects of the combination of the two. This study aimed to investigate the effects and underlying mechanism(s) of *Ascophyllum nodosum* extract, *Camellia sinensis-leaf* extract, and their joint interventions on weight loss and glycolipid metabolism in high-fat diet-induced (HFD-induced) obese mice.

## 2. Materials and methods

### 2.1. *Ascophyllum nodosum* extract and *Camellia sinensis*-leaf extract

SE and TE were provided by Beijing *Yili* Technology Development Co., Ltd. (Beijing, China). Both were extracted using an aqueous solution. The fresh materials were sequentially subjected to aqueous extraction, screening, decantation, ultrafiltration, pre-sterilization, spray drying, and homogenization to obtain dry powders. The extraction ratio of each extract was 7:1. The active ingredients are iodine (100–330 mg/kg, ICP/MA), α-amylase inhibition (≥50%, Bernfeld method), lipase inhibition (≥50%, ACS eighth ed.), minerals (≥50%/raw matter, JORF method), and tannins (≥35%/raw matter, Ph Eur.6.3 [2.8.14]/phloroglucinol method) in SE, and polyphenols (≥50%, UV-VIS method) in TE. The recipe groups are shown in Table 1.

**Table 1:** Intervention ingredients and formulas.

**Group**	**Test substance**	**Recommended dose for human [mg/(kg·d)]**	**Intervention dose in mice [mg/(kg·d)]**	**Gavage concentration [mg/mL]**
ND	Normal saline	——	——	——
HFD	Normal saline	——	——	——
SE	*Ascophyllum nodosum* extract	26.67	266.66	13.34
TE	*Camellia sinensis-leaf* extract	26.67	266.66	13.34
S3T1	*Ascophyllum nodosum* extract	20.00	200.00	13.33
		*Camellia sinensis-leaf* extract	6.67		66.67	
S2T2	*Ascophyllum nodosum* extract	13.33	133.33	13.34
		*Camellia sinensis-leaf* extract	13.33		133.33	
S1T3	*Ascophyllum nodosum* extract	6.67	66.67	13.33
		*Camellia sinensis-leaf* extract	20.00		200.00	

### 2.2. Ethics approval statement

This study conforms to the Institutional Animal Care and Use Committee (IACUC) at Xiamen University and complies with guidelines described in the Guide for the Care and Use of Laboratory Animals (Natl., Institutes of Health, Bethesda, MD, USA). The protocols for animal use were reviewed and approved by the Animal Ethical and Welfare Committee of the Laboratory Animal Center of Xiamen University (Approval No. XMULAC20200185).

### 2.3. Animal experiments

The mice (C57BL/6J male mice (*n* = 192) with an average body weight of 20.38 ± 0.87 g) were reared at 22°C, 10%−60% humidity, 12-h/12-h light–dark cycle, with free access to water. After adaptive feeding, 12 mice (ND group) were selected randomly and fed normal chow (Beijing Keao Xieli Feed Co., Ltd.; Beijing, China) for 8 weeks. The remaining mice were fed a high-fat diet (Research Diets, Inc.; Suzhou, China) for 8 weeks to establish obesity models. Mice displaying a 40% increase in body weight after a high-fat diet were considered successful obesity models and were randomly divided into one model control group (HFD) and five intervention groups, with 12 mice in each group. The sample size calculation was performed according to Mera et al. (15). The success rate of the high-fat-fed obesity model was ~40% (16), from which the initial number of animals purchased was calculated.

After successful modeling, the intervention period was officially started. All six groups continued to be fed a high-fat diet, except for the ND group. The SE and TE doses used in the intervention groups are shown in Table 1. The animal intervention doses were set at 10 times the recommended human doses according to the guidance on human and animal dose conversion (17), which was based on the approximate conversion of body surface area. The mice were administered via gavage, and the gavage volume was set at 0.1 mL/10 g body weight. The ND and HFD groups were gavaged daily with normal saline, while the rest groups were gavaged daily with the relevant plant extract solution. The intervention dose per mouse/group was always the same (0.1 mL/10 g bw), although the absolute volume of the gavage would have varied as it was calculated based on the body weight.

The body weight and feed intake per cage were measured weekly, and the energy intake was calculated based on food intake. Fasting blood glucose (FBG) levels were measured at weeks 0, 4, 8, and 10. Ten mice from each group were randomly selected for oral glucose tolerance tests (OGTTs) at the end of the intervention: After 12-h fasting, the mice were immediately administered 20% glucose solution by gavage (10 μL/g body weight). Blood was drawn from the tail vein at 0, 30, 60, and 120 min after glucose administration to measure the blood glucose concentration using a blood glucose meter (Sanuo Bioscience Co. Ltd.; Changsha, China).

### 2.4. Monitoring of animal metabolism

At the end of the intervention, four mice were randomly selected from each group and monitored using the TSE PhenoMaster (12-channel) system, which measured the respiratory entropy metabolism and voluntary movement of mice for 5 days. Each mouse was caged independently, feeding *ad libitum*. ND mice were still fed a normal diet, whereas the other groups were fed a high-fat diet. After the adaptation period (the first 48 h), stable metabolic monitoring was initiated at 0:00 and ended at 0:00 the next day. One data point was recorded every 5 min for each mouse.

The respiratory exchange ratio (RER) and energy expenditure (EE_1_) were calculated based on the O_2_ consumption and CO_2_ production measured in the exhaust gas of each cage (CO_2_ exhaled/O_2_ inhaled). Body weight was used to correct EE_1_ and obtain EE_2_, and lean body mass was used to correct EE_2_ and obtain EE_3_.

### 2.5. Tissue sampling and index testing

After 10 weeks of interventions, the mice were made to fast for 12 h. Each mouse was anesthetized with 4% isoflurane in a closed anesthetic box and removed eyeballs for blood collection. The mice were then immediately executed using cervical dislocation. The body fat (sum of peri-testicular and perirenal fat) and liver tissues were rapidly collected. The blood was centrifuged at 2,000 rpm (382 g) and 4°C for 15 min, and the supernatant was collected to measure biochemical indicators. All samples were frozen at −80°C for later use.

#### 2.5.1. Lipid body ratio (18)


$$\begin{array}{l} \text{lipid body ratio(}\%\text{) =}\frac{\text{eritesticular fat mass (g) + perirenal fat mass (g)}}{\text{body mass (g)}\times \text{100}\%\;} \end{array}$$

#### 2.5.2. Blood Lipids

Serum was collected using an automatic biochemical analyzer (Mindray BS-220) and supporting kits to detect total cholesterol (TC), triglycerides (TGs), low-density lipoproteins (LDLs), and high-density lipoproteins (HDL).

#### 2.5.3. Serum Insulin (INS), Leptin (LEP), Adiponectin (ADP), IL-1β, and TNF-α

Serum was taken to measure INS, LEP, ADP, IL-1β, and TNF-α levels in mice using a mouse enzyme-linked immunosorbent assay kit (ELISA). The insulin resistance index (homeostatic model assessment of insulin resistance, HOMA-IR) and islet β cell function (homeostatic model assessment of insulin receptor β, HOMA-β) were calculated according to the following formula (19, 20).


$$\begin{array}{l} HOMA-IR=\frac{INS\;\left(mUI/L\right)\times FBG\;\left(mmol/L\right)}{22.5}\\ HOMO-\beta =\frac{20\times INS\;\left(mUI/L\right)}{FBG\;\left(mmol/L\right)-3.5} \end{array}$$

#### 2.5.4. Hepatic lipid metabolism-related factors

Liver tissue was homogenized and centrifuged at 3,000 rpm for 10 min. The supernatant was used to measure the expression of lipoprotein lipase (LPL), fatty acid synthase (FAS), sterol regulatory element-binding transcription factor (SREBP-1c), and cholesterol-activated 7α-hydroxylase (CYP7A1) protein in the liver tissue by ELISA.

#### 2.5.5. 16S rDNA sequencing

Murine fecal samples were collected the day before the end of the experimental period and stored at −80°C. HiPure Stool DNA Kits (Magen, Guangzhou, China) were used to extract total DNA from these samples, after which the conserved 16S rDNA region (V3: 341F, CCTACGGGNGGCWGCAG; V4: 806F, GGACTACHVGGGTATCTAAT) was amplified by PCR (94°C for 2 min; 30 cycles of 98°C for 10 s, 62–66°C for 30 s [55°C for 30 s for 16S V4], 68°C for 30 s, and 68°C for 5 min) using appropriate primers and barcodes. The resultant amplicons were extracted using 2% agarose gel electrophoresis. They were purified with AMPure XP Beads (Beckman Agencourt, USA) based on provided directions, then quantified with an ABI Step One Plus Real-Time PCR System. Equimolar amounts of these amplicons were pooled and subjected to paired-end sequencing (PE250) on an Illumina instrument (Life Technologies, CA, USA) using standard protocols. Raw reads were filtered to remove low-quality reads, assembled, and filtered using FASTP (v. 0.18.0). Clean tags were clustered into operational taxonomic units (OTUs) at a ≥97% similarity threshold using the UPARSE pipeline (v. 9.2.64). The UCHIME algorithm was employed to remove chimeric tags, and the remaining tags were subjected to downstream analyses. The most abundant sequence within each OTU was selected as a representative sequence. Following OTU determination, gut microbiota analyses were conducted, including community composition, alpha diversity, and beta-diversity. Taxonomical abundance statistics were visualized using Krona (v 2.6), with R being used to analyze dominant bacteria at the phylum and genus levels. Species comparisons among groups were performed using Tukey's HSD test, and the Kruskal–Wallis H-test. QIIME (v. 1.9.1, University of Colorado, CO, USA) was used to assess changes in the overall microbiota community composition in the different groups. Principal coordinate analysis (PCoA) was conducted using the R Vegan package (v 2.5.3) and plotted using the ggplot2 package (v. 2.2.1).

### 2.6. Statistical analysis

The data were analyzed using IBM SPSS Statistics (version 26.0, SPSS Inc., USA). Descriptions are expressed as means, medians, standard deviations, and percentiles. Repeated-measures data were analyzed using the body weight, FBG, and OGTT values of the mice obtained for variance using a multivariate variance. If the data distribution was normal and the variance was homogeneous, a one-way analysis of variance (one-way ANOVA) was used to determine the variation between groups of one-way measurement indicators, and the LSD method was used for pairwise comparisons between groups. If the data distribution did not satisfy the normal distribution, the non-parametric Kruskal–Wallis H-test was used for comparison between groups, and the Nemenyi method was used for pairwise comparison of the overall mean between groups. The hypothesis test level was α = 0.05.

## 3. Results

### 3.1. Feed intake and body weight

**Figure 1:**
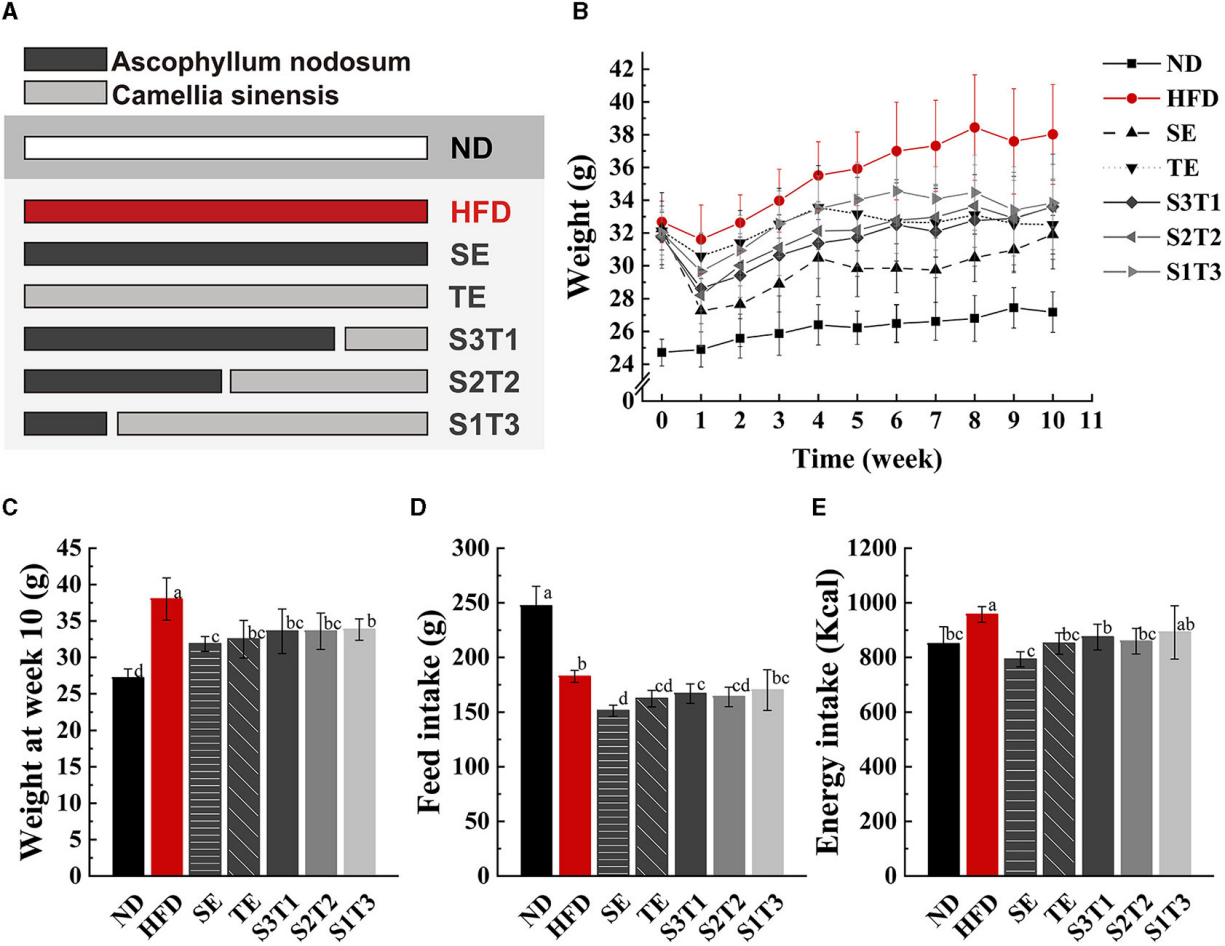
SE (*Ascophyllum nodosum* extract), TE (*Camellia sinensis*-leaf extract), and their joint interventions slowed weight gain and reduced food intake in HFD-induced obese mice. **(A, B)** Body weight changes of mice in different groups from week 0 to week 10 during the intervention period. The lines of different colors or types represent the ND (normal diet) group, HFD (high-fat diet) group, SE group, TE group, S3T1 (SE/TE = 3:1) group, S2T2 (SE/TE = 2:2) group, and S1T3 (SE/TE = 1:3) group, respectively. *n* = 12 biologically independent mice. **(C)** Body weight of mice at the end of the intervention. *n* = 12 biologically independent mice. **(D** Total feed intake per mouse during the 10-week intervention period. *n* = 5 independent cages with 2 to 3 mice in each cage. **(E)** Total energy intake per mouse during the 10-week intervention period. Data are expressed as means ± SD, and the statistical differences were analyzed by one-way ANOVA. The totally different letters indicate a significant difference (*P* < 0.05) between groups. EX: [a vs. b], [ab vs. cd], [ab vs. c]: *P* < 0.05; [a vs. a], [a vs. ab], [ab vs. b]: *P* > 0.05.

The average body weights of the HFD mice and ND mice were significantly different at week 0, being 32.68 ± 1.27 g and 24.72 ± 0.82 g (*P* < 0.001). After random grouping, no significant difference in body weight was observed among the obese mice groups (*P* > 0.05). Except for the S1T3 group, all interventions lowered food intake in mice, with SE exhibiting the most obvious appetite suppression (*P* > 0.05, Figure 1D). The results showed an interaction between the intervention duration and groups of either SE, TE, or joint interventions, which affected the body weights of the mice. Compared with the HFD group, all plant extract interventions resulted in slower weight gain in animals. Intervention with a higher SE proportion, such as in the SE, S3T1, and S2T2 groups, resulted in earlier statistical differences in body weights compared to the HFD group (≥1 week, *P* < 0.01). After 1 week, the obese mice rebounded in weight in each group. The presumption is that the previous reduction in body weight reduced the volume of the gavage and brought it down to a volume that was more adaptable to the mice. Additionally, the SE itself has a natural seaweed odor, which is not a familiar odor to rodents. After a week, the mice gradually accepted the odor and regained their appetite. At the end of week 6, the differences in weight in all the plant extract groups were statistically significant (*P* < 0.01) compared with the HFD mice. At the end of the intervention, the average body weight of each intervention group was lower than that of the HFD group (*P* < 0.05).

### 3.2. Peri-testicular and perirenal fat masses

**Figure 2:**
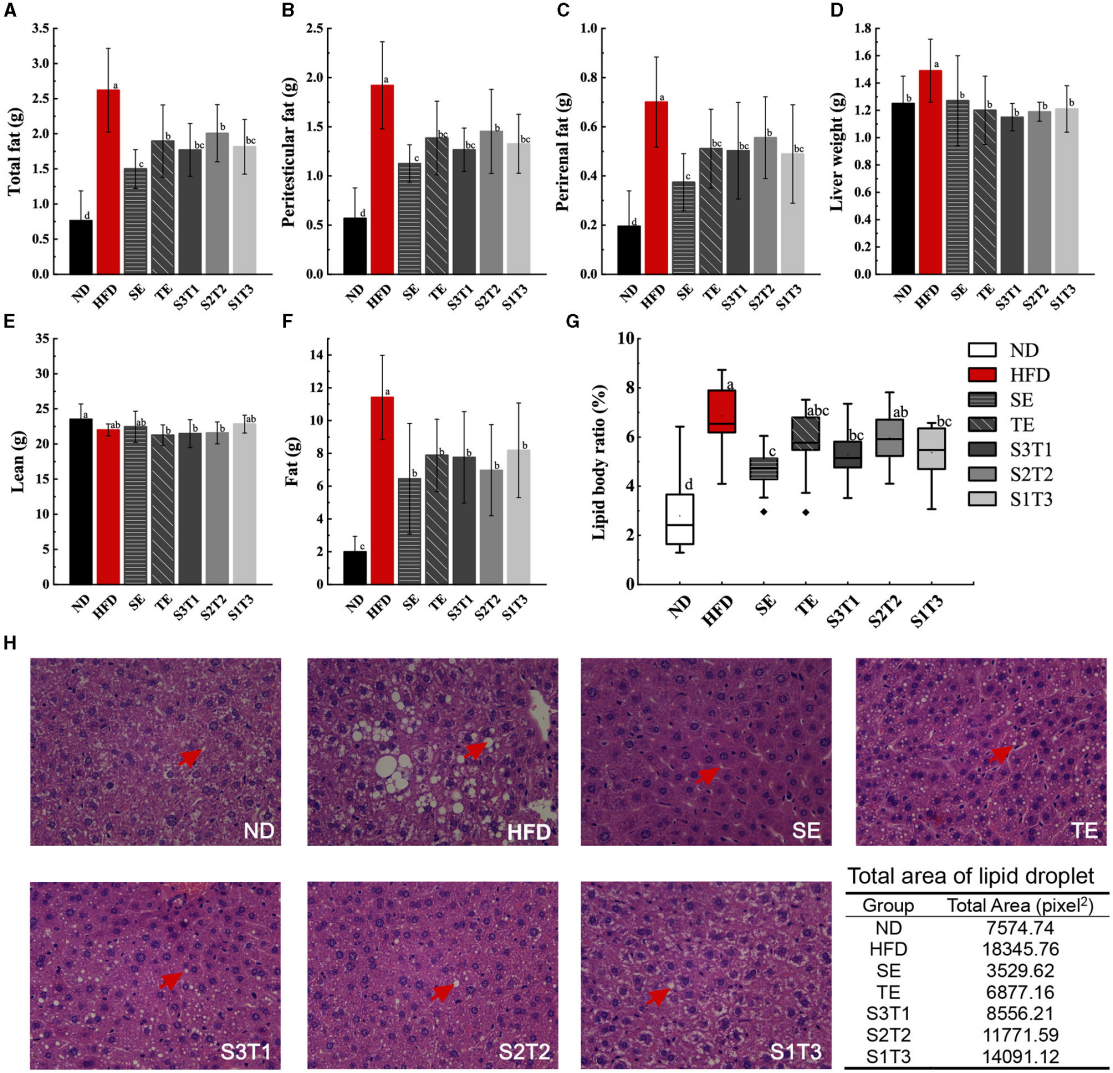
SE, TE, and their joint interventions affected the adipose tissue mass in high-fat diet-induced obese mice. **(A)** Results upon weighing adipose tissue from obese mice manually stripped during dissection. *n* = 12 biologically independent mice. **(B)** Results upon weighing peri-testicular fat tissue from obese mice manually stripped during dissection. *n* = 12 biologically independent mice. **(C)** Results upon weighing of perirenal fat tissue from obese mice manually stripped during dissection. *n* = 12 biologically independent mice. **(D)** Liver tissue weights of mice in different groups. *n* = 12 biologically independent mice. **(E)** Lean tissue mass of intervened obese mice was measured with EchoMRI-100H. *n* = 9 biologically independent mice. **(F)** Fat tissue mass of intervened obese mice was measured with EchoMRI-100H. *n* = 9 biologically independent mice. **(G)** Lipid body ratio calculated from the data in Figure 2A and body weight. **(H)** Histopathological alterations in the adipose tissue in the pathological sections. *n* = 3 biologically independent mice. Data are expressed as means ± SD or median (IQR), and the statistical differences were analyzed by one-way ANOVA or the non-parametric test Kruskal–Wallis H-test. The totally different letters indicate a significant difference (*P* < 0.05) between groups. EX: [a vs. b], [ab vs. cd], [ab vs. c]: *P* < 0.05; [a vs. a], [a vs. ab], [ab vs. b]: *P* > 0.05.

The peri-testicular and perirenal fat masses of mice in all intervention groups were lower than those in the HFD group (*P* < 0.05, Figure 2). Among the single intervention groups, SE mice had significantly lower total fat and lipid body ratios than TE mice (*P* < 0.05). Notably, single SE had a better lipid-lowering effect than combined SE. No significant difference was found in the lipid-lowering effect between the three joint interventions with different proportions of SE to TE. Similar effects were observed for the lipid body ratios calculated for each group of mice.

### 3.3. Lean and fat composition in body

The composition of lean and fat tissues in mice was used to assess the degree of body composition balance. Different diets did not significantly alter the lean tissue composition of the mice, or at least did not increase it, either with the HFD or with the interventions. However, the HFD significantly increased the fat composition of the mice (*P* < 0.05, Figures 2E, F). All interventions prevented diet-induced increases in the fat composition of obese mice (*P* < 0.05).

### 3.4. Blood lipid levels

**Figure 3:**
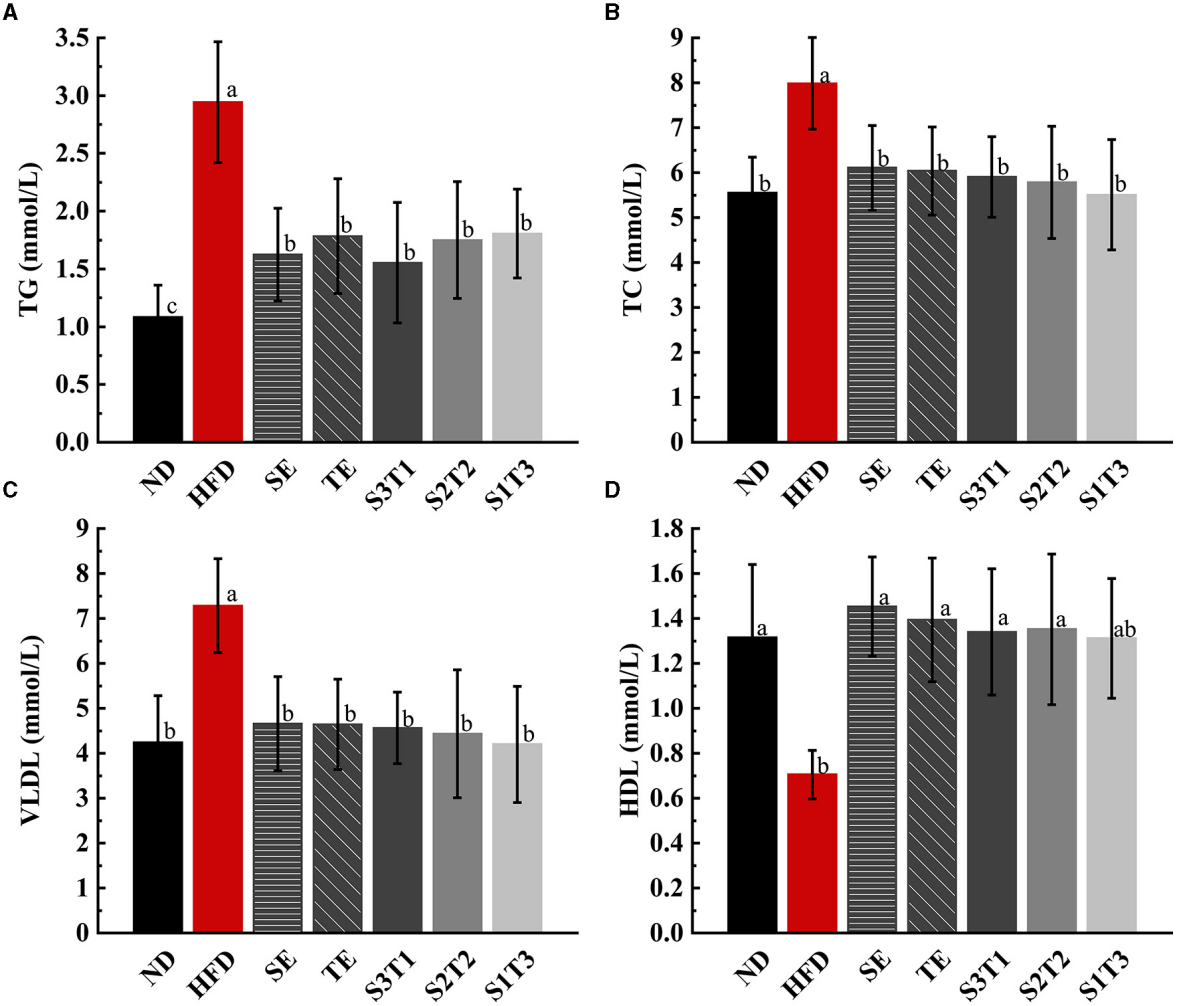
Dyslipidemia due to a high-fat diet was alleviated by SE, TE, and their joint interventions at the end of the intervention. **(A)** Triglyceride (TG) in mice serum of different groups. **(B)** Total cholesterol (TC) in mice serum of different groups. **(C)** Very low-density lipoproteins (VLDLs) in mice serum of different groups. **(D)** High-density lipoproteins (HDL) in mice serum of different groups. *n* = 8 biologically independent mice. Data are expressed as means ± SD, and the statistical differences were analyzed by one-way ANOVA. The totally different letters indicate a significant difference (*P* < 0.05) between groups. EX: [a vs. b], [ab vs. cd], [ab vs. c]: *P* < 0.05; [a vs. a], [a vs. ab], [ab vs. b]: *P* > 0.05.

HFD increased TG, TC, and LDL levels and decreased HDL in the serum of obese mice (Figure 3). Subsequently, all interventions decreased TG, TC, and LDL levels and increased HDL levels. Finally, all blood lipid indicators recovered to levels similar to those in the ND group (*P* > 0.05), except for the TG levels in some intervention groups, which remained relatively higher (*P* < 0.05 vs. the ND group). SE and TE may regulate the level of HDL, thus promoting the carriage of TC, TG, and LDL into the liver for catabolism and efflux.

### 3.5. FBG level

**Figure 4:**
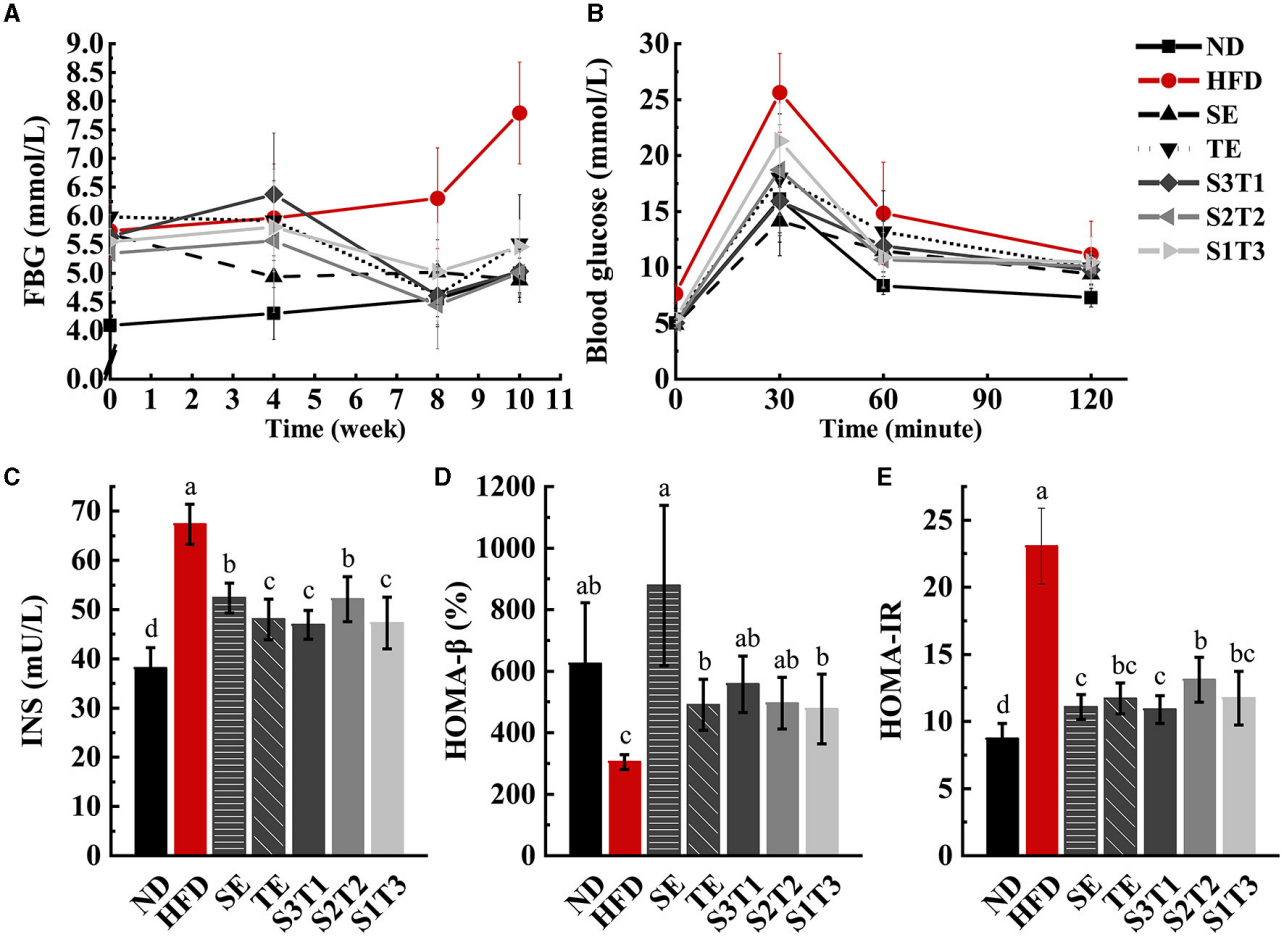
SE, TE, and their joint interventions enhanced blood glucose regulation in high-fat diet-induced obese mice. *n* = 12 biologically independent mice. **(A)** Fasting blood glucose (FBG) values in obese mice were obtained by four times repeated measurements over 10 weeks. *n* = 12 biologically independent mice. **(B)** Glucose changes in different groups were obtained during the 120-min OGTT experiment. **(C)** Plasma fasting insulin concentrations (INS) in mice at the end of the intervention. **(D)** β-cell secretion function obtained with the HOMA model. **(E)** The degree of insulin resistance obtained with the HOMA model. *n* = 8 biologically independent mice. The repeated-measures test was performed for the FBG levels and OGTT results. Data are expressed as means ± SD, and the statistical differences were analyzed by one-way ANOVA, while a repeated-measures test was performed for the FBG levels and OGTT results. The totally different letters indicate a significant difference (*P* < 0.05) between groups. EX: [a vs. b], [ab vs. cd], [ab vs. c]: *P* < 0.05; [a vs. a], [a vs. ab], [ab vs. b]: *P* > 0.05.

As opposed to the HFD group, the SE mice showed a downward trend in FBG levels, while the other groups trended toward a transient increase followed by a decrease (Figure 4A). This indicates that all interventions effectively control the HFD-induced increase in FBG levels. The FBG level in the SE group was significantly lower than that in the HFD group and only modestly higher than that in the ND group (vs. HFG: *P* < 0.05; vs. ND: *P* > 0.05) at week 4, with the same situation appearing in the other groups at week 8. However, all plant extract groups except the SE group exhibited a rebound in FBG levels compared to the ND group at week 10. Meanwhile, the FBG levels of SE mice were significantly lower than those of TE mice at week 10 (*P* < 0.05), and the FBG levels of the joint intervention groups with a higher proportion of SE were significantly lower than those of the group with a higher proportion of TE (*P* < 0.05).

### 3.6. OGTT test

The areas under the OGTT test curve (AUC) of each intervention group were smaller than those of the HFD group, indicating that all interventions enhanced the regulation of blood glucose in obese mice (Figure 4B). Obese mice treated with SE alone exhibited the highest ability to regulate the blood glucose level. At the moment of peak blood glucose, the SE mice had significantly lower blood glucose values than the TE, S2T2, and S1T3 groups (*P* < 0.05). Among the three joint intervention groups, the relative AUC values decreased as the proportion of SE increased, implying that SE controlled blood glucose more efficiently than the single TE supplement, both for the SE alone and for the mixtures.

### 3.7. Serum INS, HOMA-IR, and HOMA-β levels

HFD impaired islet cell function and triggered insulin resistance in mice, as reflected by the increased INS and HOMA-IR levels, as well as the decreased HOMA-β levels in the HFD group. INS and HOMA-IR levels in the intervention groups were significantly downregulated compared with those in the HFD group (*P* < 0.05, Figure 4), whereas HOMA-β levels returned to normal levels (*P* > 0.05). In the extract intervention groups, SE mice had significantly higher INS levels than TE mice (*P* < 0.05). Notably, HOMA-β levels in the SE group were higher than those in the TE and S1T3 groups, which have higher proportions of *Camellia sinensis-leaf* extract.

### 3.8. Indicators related to energy metabolism

The respiratory exchange ratio (RER) is regularly used to evaluate the utilization of energy-supplying substances. RER is calculated as 0.7 when the body solely uses fat as its predominant energy source. This value increases, at which point the energy-producing substance transitions from fat to carbohydrate. An RER value of 1 indicates that the body relies only on carbohydrate consumption for energy supply (21).

**Figure 5:**
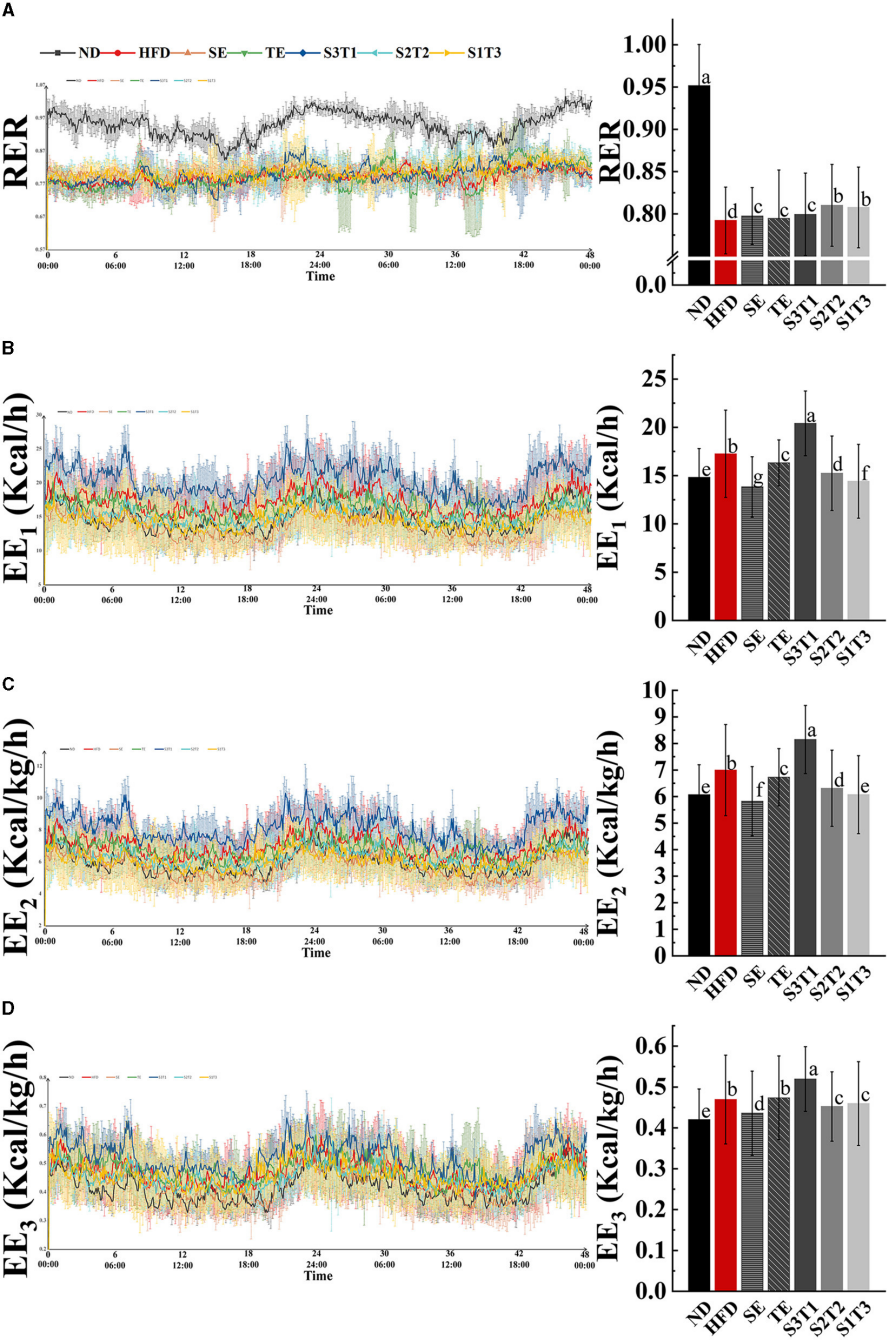
Obesity affected respiratory exchange ratio (RR) and blunted rhythm of it, whereas SE, TE, and their interventions could regulate RR and alter energy expenditure (EE) in mice. **(A)** Respiratory entropy metabolism of mice during 48 h in TSE PhenoMaster (12-channel) system. **(B)** Energy expenditure (EE_1_) metabolism of mice during 48 h. **(C)** Weight-corrected energy expenditure (EE_2_) metabolism of mice during 48 h. **(D)** Lean weight-corrected energy expenditure (EE_3_) metabolism of mice during 48 h. *n* = 4 biologically independent mice. Data are expressed as means ± SD, and the statistical differences were analyzed by one-way ANOVA. The totally different letters indicate a significant difference (*P* < 0.05) between groups. EX: [a vs. b], [ab vs. cd], [ab vs. c]: *P* < 0.05; [a vs. a], [a vs. ab], [ab vs. b]: *P* > 0.05.

RER in the ND group showed a significant rhythmic fluctuation. As expected, the RER of normal organisms was relatively higher at night than during the daytime, reaching a level close to 1.0. HFD-induced obesity, however, alters the main energy substrates of the organism, making it predominantly fat metabolizing (~0.79) and losing rhythmicity (Figure 5A). The intervention with plant extracts was not observed to have any significant alleviation of rhythm disturbances but could upregulate the RER values in mice (*P* < 0.001, intervention groups vs. HFD group, Table 2), suggesting that the interventions contributed to a shift in the body's energy metabolism from fat-based to a collaborative effort between fat and glucose. The RER levels in the S2T2 and S1T3 groups were higher than those in the SE alone and TE alone groups, indicating a synergistic effect of SE and TE in regulating respiratory entropy.

**Table 2:** Energy metabolism and respiratory entropy in mice within 48h after the intervention, $\bar{x}$ **±*s***.

**Group**	**EE_1_ (Kcal/h)**	**EE_2_ [Kcal/(kg·h)]**	**EE_3_ [Kcal/(kg·h)]**	**Food intake (g/day)**	**Exercise (m/day)**
ND	14.82 ± 2.98^e^	6.07 ± 1.13^e^	0.42 ± 0.08^e^	3.52 ± 0.41^a^	1691.07 ± 588.72^ab^
HFD	17.26 ± 4.52^b^	7.00 ± 1.71^b^	0.47 ± 0.11^b^	1.87 ± 0.89^b^	1270.12 ± 298.84^ab^
SE	13.83 ± 3.12^g^	5.82 ± 1.30^f^	0.44 ± 0.10^d^	2.08 ± 0.49^b^	1690.82 ± 754.41^ab^
TE	16.32 ± 2.38^c^	6.73 ± 1.08^c^	0.47 ± 0.10^b^	2.68 ± 1.41^b^	1195.22 ± 229.20^b^
S3T1	20.41 ± 3.35^a^	8.15 ± 1.28^a^	0.52 ± 0.08^a^	1.47 ± 0.39^b^	1409.48 ± 375.72^ab^
S2T2	15.24 ± 3.86^d^	6.31 ± 1.44^d^	0.45 ± 0.08^c^	2.14 ± 0.25^b^	1214.86 ± 137.72^b^
S1T3	14.41 ± 3.82^f^	6.07 ± 1.47^e^	0.46 ± 0.10^c^	1.55 ± 0.62^b^	1706.82 ± 282.5^a^

^1^ Values in the same column with totally different superscript letters are significantly different, with a *P*-value of ≤ 0.05 (with LSD's adjustment).

Body energy expenditure (EE) is influenced by resting energy consumption, food thermogenic effects, and activity energy consumption. Both normal-weight and obese mice had rhythmic energy expenditures and peaked at night, as shown by EE_1_, EE_2_, and EE_3_ line graphs over time. However, HFD-induced obesity was considered to increase resting energy consumption through weight gain, as evidenced by a higher energy expenditure in the HFD group mice, while exercise was significantly decreased (Table 2). This revealed the large effect of resting energy consumption on energy expenditures.

The results varied between different interventions. Energy expenditure capacity is frequently reduced by the loss of fat and lean tissue (SE group mice) or decrease reduction in physical activity (HFD and TE group mice). Nevertheless, the S3T1 group had consistently higher EE_1_, EE_2_, and EE_3_ than the other groups, despite having a lower body weight than the HFD group, as evidenced by an increase in exercise and a decrease in food intake. This implies that S3T1 might be able to increase energy expenditure by enhancing basal metabolic capacity.

### 3.9. Serum inflammatory factors IL-1β, TNF-α

IL-1β (22) and TNF-α (23) are two typical pro-inflammatory factors. Previous studies have shown that obese individuals have elevated IL-1β and TNF-α levels, which can induce insulin resistance, promote FFA production, and affect glucose and lipid metabolism (24, 25).

**Figure 6:**
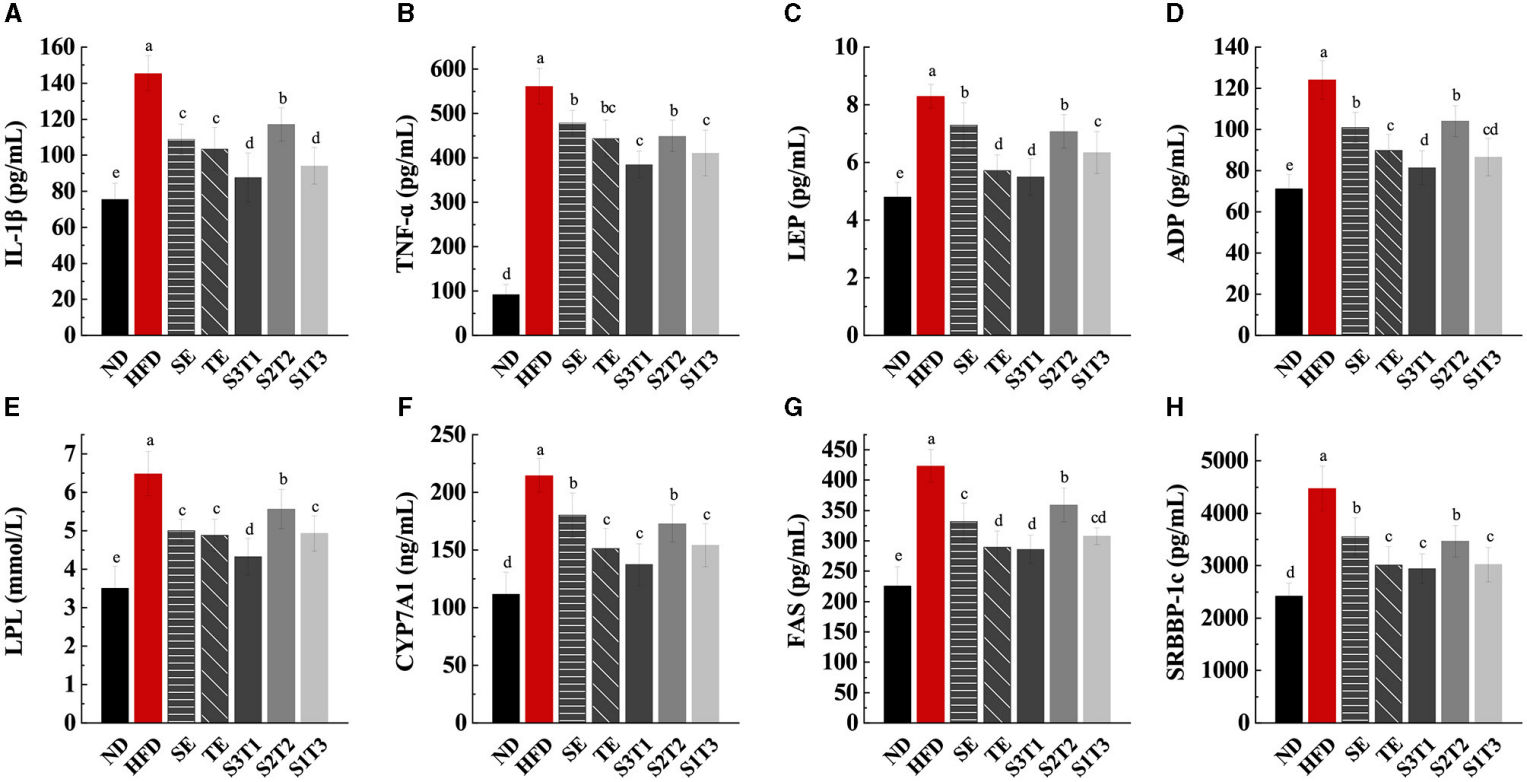
SE, TE, and their joint interventions suppressed inflammatory factor expression, leptin and lipocalin secretion, and enzyme expressions in livers involved in lipid anabolism. **(A)** IL-1β levels in mice serum at the end of the interventions. **(B)** TNF-α levels in mice serum at the end of the interventions. **(C)** LEP levels in mice serum at the end of the interventions. **(D)** ADP levels in mice serum at the end of the interventions. *n* = 8 biologically independent mice. **(E)** Lipoprotein lipase (LPL) levels in mice livers at the end of the interventions. **(F)** Cholesterol-activated 7α-hydroxylase (CYP7A1) levels in mice livers at the end of the interventions. **(G)** Fatty acid synthase (FAS) levels in mice serum at the end of the interventions. **(H)** Sterol regulatory element-binding protein-1c (SRBBP-1c) levels in mice serum at the end of the interventions. *n* = 8 biologically independent mice. Data are expressed as means ± SD, and the statistical differences were analyzed by one-way ANOVA. The totally different letters indicate a significant difference (*P* < 0.05) between groups. EX: [a vs. b], [ab vs. cd], [ab vs. c]: *P* < 0.05; [a vs. a], [a vs. ab], [ab vs. b]: *P* > 0.05.

Intervention groups showed a reduction in IL-1β and TNF-α levels in serum compared with the HFD group (*P* < 0.05, Figures 6A, B). Notably, IL-1β level reduction was greater in the S3T1 group than in the other intervention groups (*P* < 0.05).

### 3.10. Serum LEP and ADP levels

The serum levels of LEP and ADP in each intervention group were lower than those in the HFD group (*P* < 0.05, Figures 6C, D). All the interventions reduced serum LEP levels and relieved leptin resistance. When compared to SE mice, TE alone resulted in a more significant lowering of LEP and ADP levels (*P* < 0.05). The secretion of LEP may partly contribute to the appetite-regulating effect, which could potentially explain the greater decrease in food intake in the SE group.

### 3.11. LPL, CYP7A1, FAS, and SREBP-1c levels in liver tissue

LPL, CYP7A1, FAS, and SREBP-1c are key enzymes and factors that regulate lipid homeostasis and metabolism in the body. As shown in Figures 6E–H, mice in the intervention groups presented lower levels of LPL, CYP7A1, FAS, and SREBP-1 in the liver tissue than HFD mice (*P* < 0.05). When compared to the results of the SE and TE groups only, TE tended to show a better ability to regulate fat metabolism than SE (*P* < 0.05), as assessed by the level of FAS, CYP7A1, and SREBP-1c activities.

### 3.12. Changes in the composition of the gut microbiota

**Figure 7:**
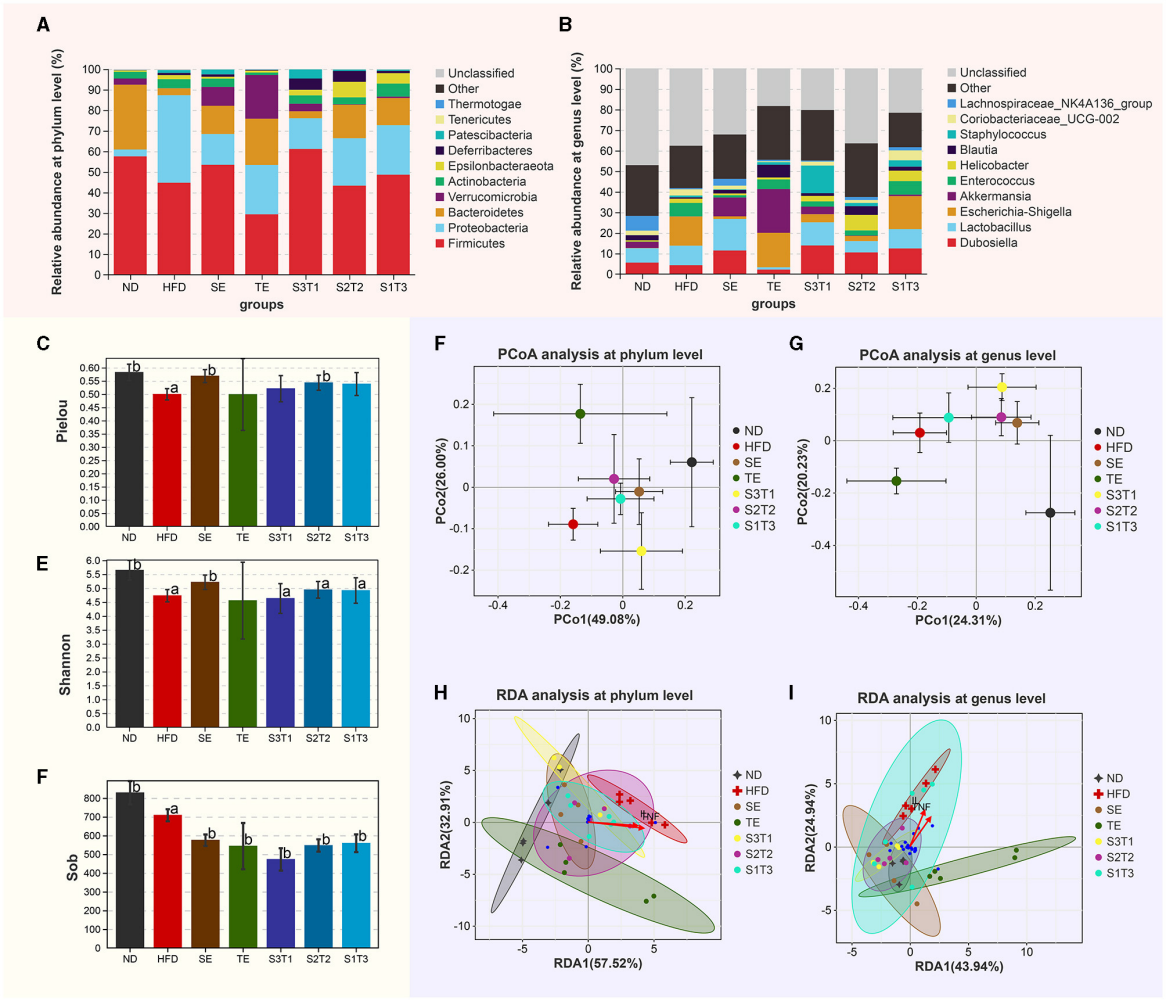
Shift of gut microbiota in each group. **(A, B)** Relative abundance of the major phyla of the gut microbiota at the **(A)** phylum and **(B)** genus levels. **(C–E)** Gavage treatment did not reverse the microbial community richness (Sob index), but somewhat improves the microbial community evenness (Pielou index) and diversity (Shannon index). **(F, G)** Principal coordinate analysis at the **(F)** phylum and **(G)** genus levels. Bray-based analysis was used to generate distance measurements between each sample. **(H, I)** The relationship between inflammatory factors and intestinal flora.

Figure 7 demonstrates the effects of different interventions on measures of alpha- (Figures 7C–E) and beta-diversity (Figures 7F–G). Bacterial richness (expressed by Sobs), diversity (expressed by Shannon index), and evenness (expressed by Pielou) were significantly reduced in HFD-induced obese mice, with the first one not being reversed by interventions with plant extracts. SE and S2T2 treatment somewhat improved the microbial community richness (Pielou index) and diversity (Shannon index). The composition of gut microbiota in each group was visualized using Bray-based principal component analysis (PCoA). PCoA plots at the phylum level revealed a separation along the direction of PCo1 (contribution of 49.08%) between HFD and ND group mice, which is dependent on the degree of obesity or high-fat diet. Treatments with plant extracts significantly altered the bacterial community in the intestine. The SE group was significantly separated from the HFD mice in the direction of PCo1 and tended toward the ND mice, as was observed in the groups with different proportions of seaweed extracts. The TE intervention did not appear to cause a significant shift in PCo1, but the shift was reflected in PCo2 (contribution of 26.00%). The SE and S3T1 groups were located on the same side of the ND group in the PCo1 direction, while the TE and S2T2 groups were located on the same side of the ND in the PCo2 direction. PCoA plots at the genus level showed a similar clustering pattern. Interestingly, the higher the SE proportion in the interventions, the closer the bacterial community of mice aggregates to the ND group.

The relative abundance of the major phyla present in the gut of different groups after treatments is depicted in Figures 7A, B. (1) At the phylum level, compared with the ND group, HFD mice had a significantly lower abundance of *Bacteroidetes* (*P* < 0.05), and a higher abundance of *Proteobacteria* (*P* < 0.001) and *Patescibacteria* (*P* < 0.01). The relative abundance of *Proteobacteria* was significantly reduced after plant extract interventions compared to HFD mice (*P* < 0.001). SE, TE, and their joint interventions increased the relative abundance of *Bacteroidetes* and *Verrucomicrobia* and depressed the F:B ratio. Due to several individual instabilities, the dominant flora in the S3T1 group of obese mice failed to be significantly reversed and remained similar to the HFD group. (2) At the genus level, *Enterococcus, Escherichia–Shigella, Helicobacter, Staphylococcus, Mucispirillum, Candidatus_Saccharimonas, Enterorhabdus, Rikenella* etc., which were positively correlated with obesity, were significantly increased in the HFD mice, whereas the relative abundances of *Roseburia, Intestinimonas*, and *Oscillibacter* were lower than the ND group (*P* < 0.05). When compared with the HFD group, the SE treatment increased the percentage of not only *Intestinimonas, Oscillibacter* but also *Dubosiella, Lactobacillus*, and *Bacteroides*, and reduced *Enterococcus, Escherichia–Shigella*, and *Helicobacter*. The TE treatment increased the percentages of *Akkermansia, Blautia, Alistipes*, and *Bacteroides* while decreasing *Prevotella* and reducing *Coriobacteriaceae_UCG-002*.

In the RDA analysis (Figures 7H, I), inflammatory indicators such as TNF-α and IL-1β influenced species distribution. The abundance of the *Proteobacteria* was positively correlated with the concentration of those inflammatory factors, whereas the abundance of the *Bacteroidetes* and *Firmicutes* were negatively correlated with them. Furthermore, at the genus level, these inflammatory indicators had a positive correlation with *Escherichia–Shigella* and *Enterococcus*, with the highest abundance in HFD. The bacterial taxa of the HFD and ND groups separated in different directions at the phylum and genus levels, and the intestinal flora of obese mice treated with plant extracts tended to revert to the ND group. Notably, the SE and SE-containing intervention groups most closely matched the bacterial taxon distribution of ND mice. The distribution of these plant extract intervention groups all shifted away from the TNF-α and IL-1β arrows.

## 4. Discussion

This study mainly investigated the effects of *Ascophyllum nodosum* and *Camellia sinensis-leaf* extracts on metabolic homeostasis in the mouse model of HFD-induced obesity and explored the functional characteristics and synergies of the different ingredients. All mice in the intervention groups were effectively protected against the persistent body weight increase that afflicted HFD mice, even without any mandatory control of the dietary intake, suggesting that the plant extracts elicited some intrinsically beneficial alterations in the organism. Plant extracts' supplementation alleviated fat accumulation, dyslipidemia, and dysbiosis caused by HFD. Some improvements in voluntary exercise and energy metabolism were also observed. We predict that the interventions slowed down the digestion, absorption, and utilization of exogenous lipids and inhibited endogenous lipid synthesis in the liver. Meanwhile, interventions accelerated lipolysis and improved glucose metabolism by alleviating chronic inflammation and insulin resistance. Having achieved the regulation of glucolipid metabolism, enzyme level, and intestinal flora structure, the energy metabolism function of the organism was enhanced, thus achieving the control of obesity.

The interventions might attenuate the degree of digestion, absorption, and utilization of exogenous lipids. Lipid metabolism disruption caused by HFD was reflected in the high TG, TC, and LDL levels in HFD mice. This is a common manifestation often found in obesity and lipid metabolic disorder-related diseases. In addition, histopathological alterations in the adipose tissue caused by HFD could be seen in the pathological sections, as evidenced by an increase in the amount and volume of *Bacteroides* is considered to be a microorganism closely associated with the lipid absorption capacity (26). We noticed that the growth of *Bacteroides* was inhibited in the HFD group, while it was significantly restored by SE and TE interventions. Among the functional analyses of the flora, the intervention with a higher percentage of TE had the most significant reduction in lipid metabolism. This suggests that interventions might inhibit lipid digestion and absorption processes by affecting the growth of specific flora. The gut is one of the most responsive organs to changes in dietary structure as just 1 week of high-fat dietary feeding could induce the differentiation of intestinal stem cells toward lipid-absorbing epithelial cells and enrichment of transcriptional features of intestinal epithelial cells toward the lipid transport pathway (27). These suggest that high-fat diets could enhance the intestinal absorption of lipids in the short term, which also explains, to some extent, the appearance of weight gain and hyperlipidemia in HFD mice. BrdU and PAS staining are necessary to be included in subsequent studies to demonstrate that the intake of these plant extracts contributes to the altered absorption characteristics of the intestinal epithelium. Recent studies show that intestinal flora could influence lipid digestion and absorption by increasing intestinal permeability or increasing the production of SCFAs (28). Targeted metabolomics regarding SCFAs would also be helpful in illustrating changes in intestinal permeability in obese mice. HFD mice were found to have significant lipid droplet accumulation in the liver, with significantly higher serum TG, TC, and LDL levels compared to the ND group. This might be due to the burden caused by large lipid intake forcing the liver to continuously use and convert TG and FFAs, transporting them to peripheral tissues or subcutaneously in the form of lipoproteins, or there might still be continuous accumulations of TG that fail to be utilized in the blood circulation or remain in the hepatocytes, causing liver damage. Liver lipid vacuoles were effectively reduced in mice after plant extract intervention, especially in the SE group. Furthermore, the blood lipid levels of the mice were significantly lowered. Additionally, HFD mice mainly consume lipids for energy supply, as assessed by the RER results. Whereas, the proportion of lipids involved in energy supply was significantly decreased after the plant extracts interventions, which indicated that the interventions did attenuate the utilization of lipids.

The interventions could alleviate the chronic inflammation caused by HFD. Multiple cytokines could promote chronic inflammation by activating macrophages in white adipose tissue (WAT), and further cause disturbances in glucose, lipids, and energy metabolism (29, 30). Macrophage infiltration leads to LEP level changes, and the secretion of inflammatory factors is associated with ADP (31). Elevated levels of TC, LEP, and ADP were observed in the subgroup with high levels of TNF-α and IL-1β, suggesting chronic inflammation in the obese state drives disturbances in glucose and lipid metabolism (25). We also found that the interventions reduced the levels of inflammatory factors such as TNF-α and IL-1β. Moreover, IL-1β promotes the release of inflammatory cytokines by mediating islet β-cell apoptosis or activating the NF-κB pathway, thereby inducing insulin resistance and type 2 diabetes (24, 32). TNF-α participates in the apoptosis and atrophy of brown adipocytes induced by insulin resistance and promotes the development of obesity with hyperinsulinemia (23, 33, 34). Evidently, the development of chronic inflammation can trigger a series of negative changes related to metabolism. The plant extract interventions reduced serum levels of inflammatory factors such as TNF-α and IL-1β, as well as levels of other biomarkers related to islet function and lipid metabolism function. The interventions increased the RER values, which implied an increase in glucose as a substrate of energy metabolism, likely related to their ability to alleviate insulin resistance by reducing IL-1β and TNF-α levels.

The interventions might inhibit liver lipid synthesis while accelerating oxidative lipolysis. Increased levels of SREBP-1c and FAS in HFD mice might be an overexpression driven by endoplasmic reticulum stress through the regulation of the PERK-SREBP-1c-FAS signaling pathway, triggered as a result of chronic low-grade inflammation (35, 36). SE and TE interventions alone or in combination reduced FAS and SREBP-1c levels in obese mice. The downregulation of the expression of these key enzymes, which regulate lipid synthesis, might have contributed to the reduced peri-testicular and perirenal fat mass in mice. Additionally, abnormal insulin secretion can also affect the rate of lipolysis reactions (37). The β-cell function, glucose tolerance, and blood lipid levels of mice in the plant extract intervention groups were significantly restored to normal levels. Behind this might be the enhanced ability to uptake and utilize glucose, which inhibits lipid synthesis and promotes lipid mobilization following a gradual decline in insulin secretion.

The regulation of glucose and lipid metabolism is directly related to the status of energy metabolism. Interestingly, HFD-induced obesity significantly altered the RER of the mice, not only in terms of a decrease in the values but also in terms of a disruption of the rhythm in respiratory entropy. RER could characterize the variation in energy metabolism in the body. Moreover, the circadian rhythm of adipose tissue lipid metabolism plays a critical role in the development of hyperphagia and obesity (38). Our findings are similar to those of Hatori et al., in that high-fat feeding blunted the rhythmicity of the RER (39). Although plant extracts failed to restore the RER rhythmicity, they elevated its absolute value, which meant that glycolipid metabolism moved to a more balanced level. The interventions of SE, S3T1, and S1T3 could increase energy expenditure by establishing a good mental state and enhancing physical vitality, which could have a weight control effect. ADP and LEP are also biomarkers associated with energy metabolism. We found that the LEP of the HFD mice in this study was significantly higher than that of the ND mice, which may cause LEP resistance, leading to a loss of the brain's judgment of energy excess and eventual obesity (40, 41). The fact that HFD mice still consumed massive amounts of feed without restraint did verify this. ADP, similarly secreted by adipocytes, regulates both glycogenolysis and fat oxidation and is correlated with body mass in studies (42). ADP was downregulated in all mice supplemented with plant extracts compared to HFD mice. This indicated that adipose tissue secreted more LEP and ADP in HFD mice to regulate the imbalance in body weight or energy metabolism. Supplementation with plant extracts inhibited the activity of these regulatory enzymes, a possible consequence of the shrinkage of adipose tissue, limiting the secretion of ADP and LEP. Lower LEP levels or reduced LEP sensitivity has positive implications in the context of obesity-associated LEP resistance.

16S rDNA sequencing was employed to explore the effects of obesity, inflammation, and plant extracts on the species composition and diversity of the intestinal flora. In the context of obesity or chronic inflammation, HFD mice have a significant dysbiosis of their intestinal flora, be it in species composition, or diversity. Notably, the analysis of differential bacterial abundances in this research was based on the Kruskal–Wallis rank-sum test, which may increase the probability of obtaining false positives or false negatives in this kind of dataset. Elevated F:B values are known to drive obesity by promoting lipid uptake by the intestine and accelerating energy absorption (43). In the present study, we discovered that the intervention of plant extracts significantly reduced F:B values compared to the HFD group. Surprisingly, the relative levels of the F:B ratio among the groups were very similar to their ranking based on the level of EE values, and even the S3T1 group's high level of F:B matched its high level of EE. This finding supported the possibility of a link between F:B values and energy metabolism (44). Unsurprisingly, at the genus level, we discovered that many genera associated with obesity, such as *Escherichia–Shigella, Enterococcus, Enterorhabdus, Rikenella*, and *Helicobacter*, were significantly higher in the stools of HFD mice. The administration of SE effectively reduced the growth of some of these harmful bacteria (*Escherichia–Shigella* and *Helicobacter*) while increasing the proportion of genera such as *Roseburia, Intestinimonas*, and *Oscillibacter*. In contrast, the TE intervention boosted the growth of several other groups (*Akkermansia, Blautia, Prevotella, Alistipes*, and *Parabacteroides*). *Bacteroides* is a microorganism closely associated with lipid absorption capacity (26). Meanwhile, the metabolites produced by *Bacteroides* using mucins can promote the growth of *Akkermansia* (45). We found that both TE and SE increased the relative abundance of the *Bacteroidetes* and that the growth of *Akkermansia* in the TE group was somewhat promoted. Intervention studies have shown that tea or polyphenol-rich foods can increase the abundance of *Akkermansia* in the gut, thereby improving metabolic function in obese individuals, and our findings support this (46). *Akkermansia* and their secretions, such as extracellular vesicles (EV), can maintain intestinal homeostasis by binding to the Toll-like receptor (TLR) of colonic epithelial cells and regulating the expression of various proteins, resulting in the improvement of diseases such as high-fat diet-induced obesity and inflammatory bowel disease (47). Overall, these findings suggest that SE and TE may have a beneficial effect on intestinal microbes, contributing to the improvement of metabolic disorders.

SE alone showed a more effective capacity for fat loss and weight maintenance. The joint intervention groups also showed a trend toward better weight control with higher proportions of *Ascophyllum nodosum* extract. It is hypothesized that the weight loss effect of the *Ascophyllum nodosum* extracts is partly due to its appetite suppression, resulting in lowest food intake in the SE mice. Moreover, the regulation of blood glucose by SE may be superior to TE, as assessed by the FGB and OGTT tests. TE, on the other hand, focuses on regulating the expression of cytokines (LEP, ADP) and hormones (FAS, CYP7A1, and SREBP-1c) associated with energy metabolism. The above indicates that SE is more aggressive in decreasing blood glucose in obese mice with the addition of the *Camellia sinensis-leaf* component, making this process milder. Joint interventions, especially those with a higher proportion of *Ascophyllum nodosum* extracts, often showed better anti-inflammatory effects. In addition, S3T1 more notably regulated various key enzymes involved in lipid synthesis and metabolism; certainly, TE alone showed the same benefits in some of those indicators. S3T1 also had a better effect on regulating metabolic energy balance than a single supplement.

The SE and S3T1 groups were prominent among the groups, with the strength of SE reflected in the control of body weight and blood glucose and that of S3T1 in the alleviation of inflammation and the improvement of energy metabolism. We noticed that SE mice often consumed less food than other mice. Apart from the effect of SE on appetite-regulating hormones or enzymes such as LEP and FAS, we could not rule out the possibility of sensory discomfort caused by the bitter taste. With proper food intake, S3T1 mice performed well in terms of glucolipid metabolism and inflammation improvement. Furthermore, when examining energy metabolism, S3T1 mice showed higher EE_3_ values, despite moderate exercise and stable weight control. The higher equilibrium indices of the S3T1 group might imply that it allowed the body to obtain more beneficial effects on glucolipid metabolism, inflammation suppression, and balanced energy expenditure under milder conditions.

In conclusion, *Ascophyllum nodosum* extracts and *Camellia sinensis-leaf* extracts controlled body weight and repaired the fatty lesions of the liver cells. By measuring the levels of various biomarkers, the interventions improved obesity-related dysglycemia, alleviated chronic inflammation, and downregulated the expression of enzymes related to lipid metabolism. While balancing the lipid glucose energy supply ratio (RER), S3T1 can even increase the body's energy expenditure value. Both SE and TE promoted the growth of *Bacteroidetes* in the gut, while TE also promoted the growth of *Akkermansia*. Behind this might be the fact that the interventions slow down the digestion, absorption, and utilization of exogenous lipids by modulating the biometabolic function of the gut flora or by affecting the absorption characteristics of the intestinal epithelium. Meanwhile, the interventions maintained the balance of lipid synthesis and consumption by improving chronic inflammation and glucose metabolism and promoting lipid mobilization. Alterations in glucolipid metabolism, cytokines, enzymes, and gut flora might together affect metabolic function in obese mice. Joint supplementation with them has more internalized and systemic health benefits as assessed by energy metabolism and anti-inflammatory effects. Metabolomic analysis of SCFAs and various types of complex O-glycans in feces will be carried out in subsequent experiments. Algorithms such as DESeq2 or ALDEx2 will be used in subsequent analyses to improve the sensitivity and specificity of the examination. Further experiments are required to explore the impact of *Ascophyllum nodosum* and *Camellia sinensis-leaf* extracts on obesity, not only focusing on blood glucose, inflammatory factors, enzymes, etc. but also on the circadian rhythm of lipid metabolism and exercise joint interventions, all of which can contribute to their beneficial effects.

## Data availability statement

The original contributions presented in the study are included in the article/supplementary material, further inquiries can be directed to the corresponding author.

## Ethics statement

The animal study was reviewed and approved by the Animal Ethical and Welfare Committee of the Laboratory Animal Center of Xiamen University (Approval No. XMULAC20200185). The study was conducted in accordance with the local legislation and institutional requirements.

## Author contributions

YX was responsible for formal analysis, investigation, extracting and analyzing data, supervision, validation, and writing the original draft. XJ contributed to writing the protocol and report, supervision, extracting and analyzing data, and visualization. WZ was responsible for data curation, validation, and writing-reviewing and editing. QX conducted the data analysis, formal analysis, and writing-original draft. MZ contributed resources and data curation. ZZ, JHa, HaL, JD, YL, and HF provided feedback on the report. JHe was responsible for validation, supervision, and writing-review and editing. HoL was responsible for conceptualization, funding acquisition, project administration, and writing-reviewing and editing. All authors contributed to the article and approved the submitted version.

## References

[1] Maruvada P Leone V Kaplan LM Chang EB. The human microbiome and obesity: moving beyond associations. Cell Host Microbe. (2017) 2:588–99. 10.1016/j.chom.1005 29120742

[2] Null R Consultation W. Obesity: Preventing and Managing the Global Epidemic. Geneva: World Health Organization. (2000) 15:18–30. 11234459

[3] World Obesity Federation. A Different Scale: Global Action to Address Obesity. (2017).

[4] World Health Organization. Draft Recommendations for the Prevention and Management of Obesity over the Life Course, Including Considering the Potential Development of Targets in This Regard. In, Annex. (2022) 9, 104.

[5] Blunt JW Copp BR Munro MH Northcote PT Prinsep MR. Marine natural products. Royal Soc Chem. (2005) 5:124. 10.1039./c6np00124f 15692616

[6] Shin HC Kim SH Park Y Lee BH Hwang HJ. Effects of 12-Week Oral Supplementation of ecklonia cava polyphenols on anthropometric and blood lipid parameters in overweight korean individuals: a double-blind randomized clinical trial. Phytotherapy Res. (2012) 26:363–8. 10.1002./ptr.3559 21717516

[7] Vodouhè M Marois J Guay V Leblanc N Weisnagel SJ Bilodeau JF. Marginal impact of brown seaweed ascophyllum nodosum and fucus vesiculosus extract on metabolic and inflammatory response in overweight and obese prediabetic subjects. Mar Drugs. (2022) 20:174. 10.3390/md20030174 35323474 PMC8951415

[8] Catarino MD Amarante SJ Mateus N Silva AM Cardoso SM. Brown algae phlorotannins: a marine alternative to break the oxidative stress, inflammation and cancer network. Foods. (2021) 7:3559. 10.3390/foods10071478 34202184 PMC8307260

[9] Catarino MD Silva AM Mateus N Cardoso SM. Optimization of phlorotannins extraction from fucus vesiculosus and evaluation of their potential to prevent metabolic disorders. Mar Drugs. (2019) 17:162. 10.3390./md17030162 30857204 PMC6471631

[10] Kao YH Hiipakka RA Liao S. Modulation of endocrine systems and food intake by green tea epigallocatechin gallate. Endocrinology. (2000) 141:980–87. 10.1210/endo.141.3.7368 10698173

[11] Kawser Hossain M Abdal Dayem A Han J Yin Y Kim K Kumar Saha S. Molecular mechanisms of the anti-obesity and anti-diabetic properties of flavonoids. Int J Mol Sci. (2016) 17:569. 10.3390/ijms17040569 27092490 PMC4849025

[12] Hsu TF Kusumoto A Abe K Hosoda K Kiso Y Wang MF. Polyphenol-. Eur J Clin Nutr. (2006) 60:1330–36. 10.1038/sj.ejcn.1602464 16804556

[13] Wolfram S Raederstorff D Wang Y Teixeira SR Elste V. TEAVIGO (Epigallocatechin Gallate) supplementation prevents obesity in rodents by reducing adipose tissue mass. Annals Nutri Metabol. (2005) 49:54–63. 10.1159/000084178 15735368

[14] Murase T Nagasawa A Suzuki J Hase T Tokimitsu I. Beneficial effects of tea catechins on diet-induced obesity: stimulation of lipid catabolism in the liver. Int J Obes Relat Metab Disord. (2002) 26:1459–64. 10.1038/sj.ijo.0802141 12439647

[15] Mera R Thompson H Prasad C. How to calculate sample size for an experiment: a case-based description. Nutr Neurosci. (1998) 1:87–91. 10.1080/1028415X.1998.11747217 27405915

[16] Han-ying Z Hui L Ying-ying Z Hong-wei L. Effects of bifidobacterium CP-9 and lactobacillus reuteri on glucose. Acta Nutrimenta Sinica. (2021) 43:283–88. 10.13325/j.cnki.acta.nutr.sin.0301234166387

[17] State Administration for Markrt Regulation,. (2020). Traditional Chinese Medicine on the Release of the Catalogue of Nutrient Supplements for Health Food Raw Materials (2020 Edition). 2020-1606804879208. Available online at: https://gkml.samr.gov.cn/nsjg/tssps/202012/t20201201_324005.html

[18] Li Q Liu F Liu J Liao S Zou Y. Mulberry leaf polyphenols and fiber induce synergistic antiobesity and display a modulation effect on gut microbiota and metabolites. Nutrients. (2019) 11:17. 10.3390./nu11051017 31064150 PMC6567141

[19] Matthews DR Hosker JP Rudenski AS Naylor BA Treacher DF Turner RC. Homeostasis model assessment: insulin resistance and beta-cell function from fasting plasma glucose and insulin concentrations in man. Diabetologia. (1985) 28:412–19. 10.1007/BF00280883 3899825

[20] Toin T Reynaud Q Denis A Durieu I Mainguy C Llerena C. (2022). HOMA indices as screening tests for cystic fibrosis-related diabetes. J Cystic Eur Cys Fibrosis Soc. (2021) 21:123–28. 10.1016/j.jcf.05010 34090803

[21] Weir JDV. New methods for calculating metabolic rate with special reference to protein metabolism. J Physiol. (1949) 109:1–9. 10.1113/jphysiol.1949.sp004363 15394301 PMC1392602

[22] Dinarello CA van der Meer JW. Treating inflammation by blocking interleukin-1 in humans. Sem Immunol. (2013) 25:469–84. 10.1016/j.smim.10008 24275598 PMC3953875

[23] Bouter B Geary N Langhans W Asarian L. Diet-genotype interactions in the early development of obesity and insulin resistance in mice with a genetic deficiency in tumor necrosis factor-alpha metabolism. Clin Exp. (2009) 59:1065–73. 10.1016/j.metabol.11003 20045154

[24] Petrasek J Bala S Csak T Lippai D Kodys K Menashy V. IL-1 receptor antagonist ameliorates inflammasome-dependent alcoholic steatohepatitis in mice. J Clin Invest. (2012) 122:3476–89. 10.1172/JCI60777 22945633 PMC3461900

[25] Zhou L Chen H Xu Q Han X Zhao Y Song X. The effect of di-2-ethylhexyl phthalate on inflammation and lipid metabolic disorder in rats. Ecotoxicol Environ Safety. (2019) 170:391–8. 10.1016/j.ecoenv.1009 30550969

[26] Martinez-Guryn K Hubert N Frazier K Urlass S Musch MW Ojeda P. Small intestine microbiota regulate host digestive and absorptive adaptive responses to dietary lipids. Cell Host and Microbe. (2018) 23:458–69. 10.1016/j.chom.03011 29649441 PMC5912695

[27] EEnriquez JR McCauley HA Zhang KX Sanchez JG Kalin GT Lang RA. A dietary change to a high-fat diet initiates a rapid adaptation of the intestine. Cell Rep. (2022) 41:111641. 10.1016/j.celrep.2022.111641 36384107 PMC9817065

[28] Moreno-Indias I Cardona F Tinahones FJ Queipo-Ortuño MI. Impact of the gut microbiota on the development of obesity and type 2 diabetes mellitus. Front Microbiol. (2014) 5:190. 10.3389/fmicb.2014.00190 24808896 PMC4010744

[29] Larabee CM Neely OC Domingos AI. Obesity: a neuroimmunometabolic perspective. Nat Rev Endocrinol. (2020) 16:30–43. 10.1038/s41574-019-0283-6 31776456

[30] Reilly SM Saltiel AR. Adapting to obesity with adipose tissue inflammation. Nat Rev Endocrinol. (2017) 13:633–43. 10.1038/nrendo.2017.90 28799554

[31] Weisberg SP McCann D Desai M Rosenbaum M Leibel RL Ferrante AW. Obesity is associated with macrophage accumulation in adipose tissue. J Clin Invest. (2003) 112:1796–808. 10.1172/JCI19246 14679176 PMC296995

[32] Münzberg H Morrison CD. Structure, production and signaling of leptin metabolism. Clin Exp. (2014) 64:13–23. 10.1016/j.metabol.09010 PMC4267896 25305050

[33] Bastard JP Maachi M Lagathu C Kim MJ Caron M Vidal H. Recent advances in the relationship between obesity, inflammation, and insulin resistance. European Cytokine Network. (2006) 17:4–12. 16613757

[34] Uysal KT Wiesbrock SM Marino MW Hotamisligil GS. Protection from obesity-induced insulin resistance in mice lacking TNF-alpha function. Nature. (1997) 389:610–14. 10.1038/39335 9335502

[35] Wang S Tao J Chen H Kandadi MR Sun M Xu H. Ablation of Akt2 and AMPKα2 rescues high fat diet-induced obesity and hepatic steatosis through parkin-mediated mitophagy. Acta Pharmaceutica Sinica. B. (2021) 11:3508–26. 10.1016/j.apsb.07006 34900533 PMC8642450

[36] Zhang K Kaufman RJ. From Endoplasmic-Reticulum Stress to the Inflammatory Response Insight Review. Nature Publishing Group, no. (7203). (2008). 10.1038/nature07203 PMC2727659 18650916

[37] Voss TS Vendelbo MH Kampmann U Pedersen SB Nielsen TS Johannsen M. Effects of insulin-induced hypoglycaemia on lipolysis rate, lipid oxidation and adipose tissue signalling in human volunteers: a randomised clinical study. Diabetologia. (2017) 60:143–52. 10.1007/s00125-016-4126-x 27734104

[38] Paschos GK Ibrahim S Song WL Kunieda T Grant G Reyes TM. Obesity in mice with adipocyte-specific deletion of clock component Arntl. Nat Med. (2012) 18:1768–77. 10.1038/nm.2979 23142819 PMC3782286

[39] Hatori M Vollmers C Zarrinpar A DiTacchio L Bushong EA Gill S. Time-restricted feeding without reducing caloric intake prevents metabolic diseases in mice fed a high-fat diet. Cell Metabolism. (2012) 15:848–60. 10.1016/jcmet04 22608008 PMC3491655

[40] Myers MG Cowley MA Münzberg H. Mechanisms of leptin action and leptin resistance. Annu Rev Physiol. (2008) 70:537–56. 10.1146/annurev.physiol.70.113006.100707 17937601

[41] Pan H Guo J Su Z. Advances in understanding the interrelations between leptin resistance and obesity. Physiol Behav. (2014) 130:157–69. 10.1016/j.physbeh.04003 24726399

[42] Wang ZV Scherer PE. Adiponectin, the past two decades. J Mol Cell Biol. (2016) 8:93–100. 10.1093/jmcb/mjw011 26993047 PMC4816148

[43] Wang S Cui K Liu J Hu J Yan K Xiao P. mogroside-rich extract from siraitia grosvenorii fruits ameliorates high-fat diet-induced obesity associated with the modulation of gut microbiota in mice. Frontiers in Nutrition. (2022) 9:870394. 10.3389/fnut.2022.870394 35769373 PMC9234556

[44] De Filippo C Cavalieri D Di Paola M Ramazzotti M Poullet JB Massart S. Impact of diet in shaping gut microbiota revealed by a comparative study in children from Europe and Rural Africa. Proceed Nat Acad Sci United States of America. (2010) 107:14691–6. 10.1073/pnas.1005963107 20679230 PMC2930426

[45] You HJ Si J Kim J Yoon S Cha KH Yoon HS. Bacteroides vulgatus SNUG 40005 restores akkermansia depletion by metabolite modulation. Gastroenterology. (2023) 164:103–16. 10.1053/j.gastro.09040 36240952

[46] Hasani A Ebrahimzadeh S Hemmati F Khabbaz A Hasani A Gholizadeh P. The role of akkermansia muciniphila in obesity, diabetes and atherosclerosis. J Med Microbiol. (2021) 70:1435. 10.1099./jmm.0.001435 34623232

[47] Cheng D Xie MZ. A review of a potential and promising probiotic candidate-akkermansia muciniphila. J Appl Microbiol. (2021) 130:1813–22. 10.1111/jam.14911 33113228

